# Resistance of the murine cornea to bacterial colonization during experimental dry eye

**DOI:** 10.1371/journal.pone.0234013

**Published:** 2020-05-29

**Authors:** Stephanie J. Wan, Sophia Ma, David J. Evans, Suzanne M. J. Fleiszig

**Affiliations:** 1 Vision Science Program, University of California, Berkeley, CA, United States of America; 2 School of Optometry, University of California, Berkeley, CA, United States of America; 3 College of Pharmacy, Touro University California, Vallejo, CA, United States of America; 4 Graduate Groups in Microbiology, and Infectious Diseases & Immunity, University of California, Berkeley, CA, United States of America; University of Florida, UNITED STATES

## Abstract

The healthy cornea is remarkably resistant to infection, quickly clearing deliberately inoculated bacteria such as *Pseudomonas aeruginosa* and *Staphylococcus aureus*. Contrasting with the adjacent conjunctiva and other body surfaces, it also lacks a resident viable bacterial microbiome. Corneal resistance to microbes depends on intrinsic defenses involving tear fluid and the corneal epithelium. Dry eye, an ocular surface disease associated with discomfort and inflammation, can alter tear fluid composition and volume, and impact epithelial integrity. We previously showed that experimentally-induced dry eye (EDE) in mice does not increase corneal susceptibility to *P*. *aeruginosa* infection. Here, we explored if EDE alters corneal resistance to bacterial colonization. EDE was established in mice using scopolamine injections and dehumidified air-flow, and verified by phenol-red thread testing after 5 and 10 days. As expected, EDE corneas showed increased fluorescein staining versus controls consistent with compromised epithelial barrier function. Confocal imaging using *mT/mG* knock-in mice with red-fluorescent membranes revealed no other obvious morphological differences between EDE corneas and controls for epithelium, stroma, and endothelium. EDE corneas were imaged *ex vivo* and compared to controls after alkyne-functionalized D-alanine labeling of metabolically-active colonizing bacteria, or by FISH using a universal 16S rRNA gene probe. Both methods revealed very few viable bacteria on EDE corneas after 5 or 10 days (median of 0, upper quartile of ≤ 1 bacteria per field of view for each group [9–12 eyes per group]) similar to control corneas. Furthermore, there was no obvious difference in abundance of conjunctival bacteria, which included previously reported filamentous forms. Thus, despite reduced tear flow and apparent compromise to corneal barrier function (fluorescein staining), EDE murine corneas continue to resist bacterial colonization and maintain the absence of a resident viable bacterial microbiome.

## Introduction

Resident microbial communities (microbiomes) exist on most mucosal surfaces and play an important role in maintaining tissue homeostasis. Remarkably, the murine cornea is devoid of a resident viable bacterial microbiome despite being constantly exposed to the environment [1]. Its neighboring tissue, the conjunctiva, supports a population of resident bacteria (in mice and in humans), albeit in fewer numbers compared to other mucosal surfaces, and these microbes can contribute to ocular surface defense against infection [1–3]. Resistance of the murine cornea to microbial colonization involves IL-1R and MyD88 [1], but multiple intrinsic defenses likely contribute. These include; physical removal by tear fluid and eye-lid blinking [4], antimicrobial properties of tear fluid [5], antimicrobial peptides expressed by ocular surface epithelia [6–8], epithelial barrier function [9] and other components of innate immunity [10, 11]. Ultimately, resistance to bacterial colonization is likely an important component of the cornea maintaining a clarity critical for vision.

Dry eye is a multifactorial disease of the ocular surface characterized by loss of tear film leading to symptoms of discomfort, inflammation and damage to the ocular surface [12]. Dry eye disease (DED) is associated with several factors that could compromise corneal defenses against microbial colonization. These include; altered tear film composition with decreased antimicrobial factors [13], a loss of conjunctival goblet cells [14], and poor epithelial integrity [15]. Indeed, it has been hypothesized that bacterial colonization may contribute to ocular surface damage and inflammation observed during DED. Support for this hypothesis was derived from several observations; a) low-dose tetracycline antibiotics that can help improve DED symptoms inhibit bacterial virulence factor expression [16], b) increased dendritic cell density in various forms of DED [17], and c) upregulation of the TLR4 receptor for bacterial lipopolysaccharide in DED [18].

Few studies have looked at the presence of ocular surface bacteria in association with DED. One study used culture methods and 16S rRNA gene sequencing to look at the posterior lid margin and lower conjunctival sac of human patients with DED compared to healthy controls [19]. Conventional culture showed a slight increase in overall bacterial numbers in severe DED, but no significant difference in the number of positive PCR swabs in normal versus DED subjects [19]. Another study assessed differences in the ocular surface microbiome using 16S rRNA gene sequencing of conjunctival swabs in patients with DED associated with Sjogren’s Syndrome, but found no difference in bacterial constituents between those patients and controls [20]. Since both of these studies were conducted in human subjects, only the conjunctiva was evaluated. It is not known if DED causes any changes in the ability of microbes to associate with the human cornea.

In a previous study, we showed that experimentally-induced dry eye (EDE) did not increase murine corneal susceptibility to colonization by deliberately-inoculated *Pseudomonas aeruginosa*, and that corneal defense under EDE conditions involved surfactant protein D (SP-D) [21]. More recently, we also showed that the healthy murine cornea and conjunctiva exhibit very different microbial constituents, the cornea lacking a microbiome of viable resident bacteria, the conjunctiva hosting viable bacterial filamentous forms [1]. Here, we tested if murine EDE would compromise corneal resistance to colonization by viable environmental bacteria, or those from the skin or conjunctiva, i.e. corneal resistance to hosting a resident viable bacterial microbiome.

## Results and discussion

EDE was established in female C57BL/6J mice, and eyes imaged by confocal microscopy after using an alkyne-functionalized D-alanine (alkDala) to label metabolically-active bacteria (peptidoglycan synthesis), and fluorescent *in situ* hybridization (FISH) with a universal bacterial 16S rRNA gene probe to detect viable bacteria *in situ* independently of peptidoglycan synthesis.

**Fig 1:**
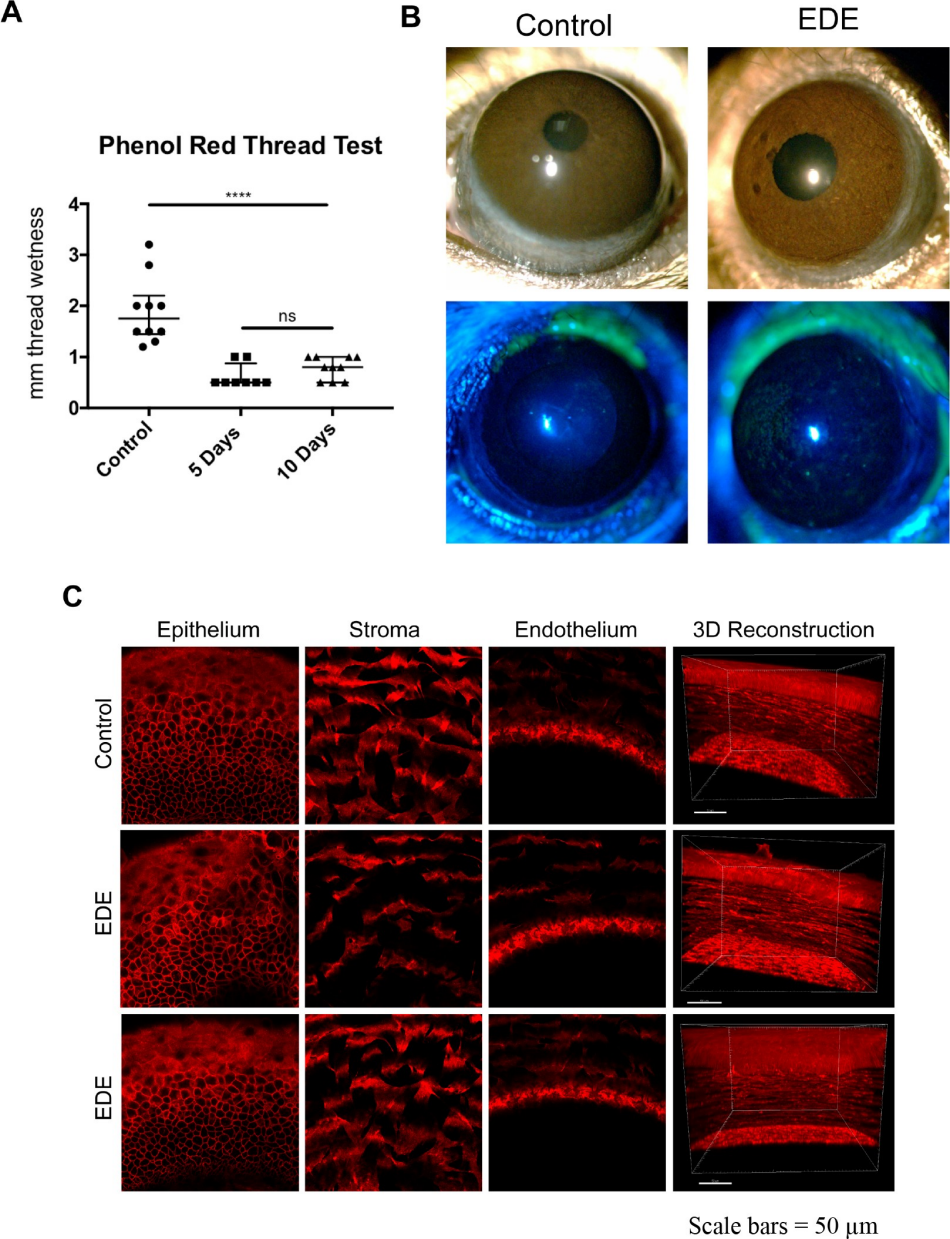
Induction of experimental dry eye. A) Tear volumes in the eyes of C57BL/6J mice under dry eye (EDE) conditions versus normal controls were measured using the phenol red thread tear test. EDE resulted in a significant decrease in tear volume after 5 and 10 days of treatment versus 10 day controls. Tears were collected from the lateral canthus using a cotton thread and reported as millimeters of wetted thread. Data are expressed as the median with lower and upper quartiles. **** = P < 0.0001, Mann-Whitney *U* test, ns = not significant (5 mice per group). B) After induction of dry eye for 10 days, representative photographs taken under the dissecting microscope demonstrate no overt changes to the ocular surface with EDE (upper panels). However, increased fluorescein staining in the EDE mice (lower panels) indicate reduced epithelial integrity. C) Transgenic C57BL/6J mice with red fluorescent cell membranes were sacrificed, then eyes enucleated and glued onto a glass cover slip and covered in DMEM to retain viability. Eyes were imaged at 0.5 μm intervals through the entire cornea. No differences in corneal morphology were detected between EDE and controls. Representative examples of the epithelium, stroma, endothelium, and a 3D reconstruction are shown. All images were taken at 10 days.

To establish EDE, mice were subjected to a 5-day or 10-day regimen of scopolamine injections and housed in dehumidified conditions (see Methods). After 5 or 10 days, aqueous tear production was assessed using phenol red thread tear tests. EDE mice showed a 54.3% decrease in tear production compared to controls after 10 days: a median of 0.5 mm thread wetness (lower quartile 0.5 mm; upper quartile 0.875 mm) at 5 days, and median of 0.8 mm (lower quartile 0.5 mm; upper quartile 1.0 mm) at 10 days versus a median of 1.75 mm (lower quartile 1.45 mm; upper quartile 2.2 mm) in 10 day controls (5 day controls were similar to 10 day controls, not shown) (Fig 1A). Increased fluorescein staining in 10 day treated mice also indicated the establishment of EDE (Fig 1B). However, those EDE mice did not exhibit any gross morphological corneal defects in the epithelium, stroma or endothelium versus controls (Fig 1C).

**Fig 2:**
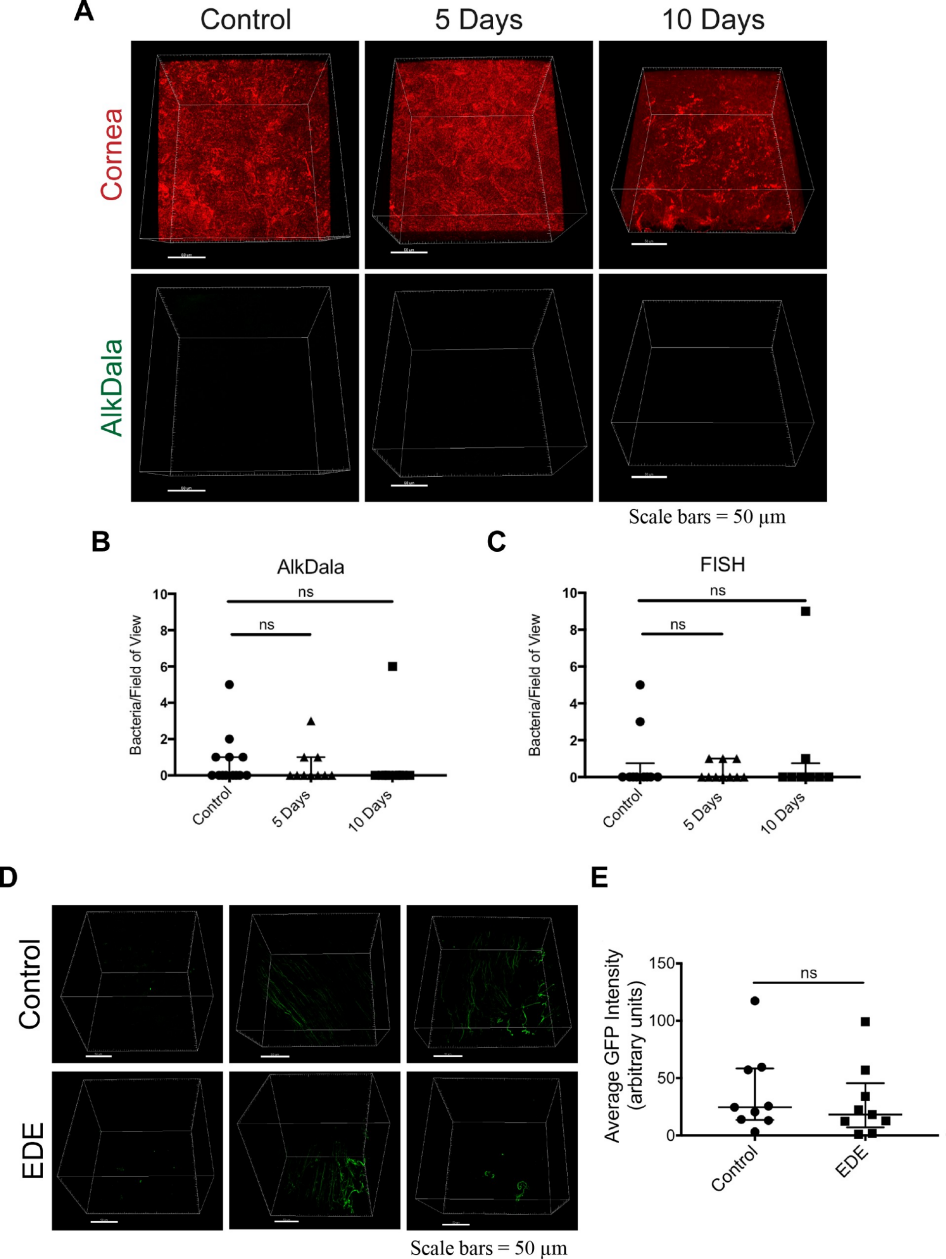
EDE did not alter the bacterial environment on the ocular surface. A) Representative confocal images of mouse corneas (upper panels) and alkDala labeling (lower panels, using same image as above with the red channel removed) in control (10 days) and EDE corneas after 5 and 10 days. Bacteria were rarely identified on the corneas in either group. B) and C) Quantification of the number of bacteria detected per field of view on the cornea of each eye imaged after AlkDala labeling (B) or FISH (C) expressed as the median with lower and upper quartiles for each group (9–12 eyes per group). Control data shown = 10 days. NS, not significant, Kruskal-Wallis test with Dunn’s multiple comparison test. D) Representative confocal images of alkDala labeling on the conjunctiva of 10 day control mice (upper panels) versus 10 day EDE mice (lower panels). E) No significant differences were detected between control and EDE mouse conjunctivae at 10 days with regard to alkDala labeling measured as average GFP intensity: control group median 24.62 (lower quartile 13.53; upper quartile 58.39) versus the EDE group median 18.18 (lower quartile 7.06; upper quartile 45.5) (9 eyes per group). NS, not significant, Mann-Whitney *U* test.

Since dry eye disease has been associated with compromise to some defense mechanisms at the ocular surface and the presence of epithelial defects, we hypothesized that EDE would enable greater bacterial colonization on the otherwise ‘colonization-resistant’ cornea. Indeed, an increased presence of bacteria could help explain inflammation and irritation associated with dry eye disease. Eyes with EDE were subjected to alkDala labeling (see Methods) to detect metabolically-active bacteria (Fig 2A). EDE mice corneas rarely had live bacteria detected: a median of 0 bacteria per field of view (upper quartile of 1) after 5 days, a median of 0 bacteria per field of view (upper quartile of 0) after 10 days both of which did not differ from control corneas with a median of 0 bacteria per field of view (upper quartile of 1) after 10 days (Fig 2B). FISH labeling using a universal bacterial 16S rRNA gene probe corroborated the results with alkDala in that viable bacteria were seldom detected on EDE mice or controls: each group showed a median of 0 bacteria per field of view (upper quartiles ≤ 1) (Fig 2C). Thus, both methods showed that EDE did not enable corneal susceptibility to colonization by viable bacteria from the environment or from neighboring tissues (i.e. skin and conjunctiva).

The abundance of viable bacteria on the conjunctiva of EDE mice and controls was also examined using alkDala labeling since previous studies using 16S rRNA gene sequencing may not have found differences in bacterial constituents on the conjunctiva due to detection of nucleic acids and not live microbes [19, 20]. Similarly, standard culture methods have significant limitations in the number and type of bacteria detected. Here, we determined if the presence of metabolically-active bacteria, including filamentous bacteria that we identified previously in the murine conjunctiva [1], differed in EDE mice versus controls. AlkDala labeling revealed numerous metabolically-active bacteria, including those of filamentous morphology, in the conjunctiva of EDE mice after 10 days, similar to controls (Fig 2D and 2E). Thus, EDE did not appear to render the conjunctival surface more susceptible to bacterial colonization. A *caveat* to this experiment, however, is that only the presence or absence of live bacteria was tested. It remains possible that dry eye disease results in changes in the species of bacteria present on the conjunctiva, as well as on the surrounding lid margins. Those changes, in turn, could influence the nature of microbial components affecting ocular surface inflammatory responses.

In summary, we used this EDE model previously to show that EDE did not compromise corneal defenses against colonization by deliberately-inoculated *P*. *aeruginosa* [21]. This study shows that EDE also does not compromise the ‘colonization-resistant’ state of the murine cornea, i.e. the absence of a resident viable bacterial microbiome. The ability of the EDE cornea to resist bacterial colonization, either from the environment or from bacterial residents of neighboring tissues, skin of the eyelids and conjunctiva, occurs despite reduced tear production, epithelial defects, and perhaps other reported compromise to ocular defenses, e.g. loss of conjunctival goblet cells [14]. These findings may reflect a functional redundancy of multiple corneal defenses in the context of EDE, and/or upregulation of others, e.g. surfactant protein-D as shown by us previously in this model [21], or defensin antimicrobial peptides [22] to compensate for EDE-induced pathological changes. Indeed, we previously showed that the absence of a resident viable bacterial microbiome on the healthy murine cornea was associated with IL-1R-dependent antimicrobial activity [1]. However, the identity of the factor(s) involved in maintaining the colonization-resistant state of the murine cornea under healthy or EDE conditions remains to be determined.

## Materials and methods

### Ethics statement

All procedures involving mice were carried out in accordance with a protocol (AUP-2016-08-9021) approved by the Animal Care and Use Committee, University of California, Berkeley which is an AAALAC accredited institution. The protocol adheres to PHS policy on the humane care and use of laboratory animals, and the guide for the care and use of laboratory animals. Procedures adhered to the ARVO Statement for the use of Animals in Ophthalmic Vision Research.

### Experimentally-induced dry eye (EDE) murine model

Six to twelve week old wild-type female C57BL/6J mice were used. In some studies, transgenic mice with fluorescent red cell membranes (*mT/mG* knock-in mice) [23] mice were used. These studies only involved female mice because in humans females have a higher incidence and severity of the dry eye disease [24], and male mice tested did not tolerate the EDE protocol requiring discontinuation of their inclusion. At the conclusion of experiments, mice were euthanized by intraperitoneal injection of ketamine (80–100 mg/Kg) and xylazine (5–10 mg/Kg) followed by cervical dislocation.

EDE was induced in mice as previously described [21]. Mice were given subcutaneous injections of scopolamine hydrobromide three times a day (0.1 mL of 10 mg/mL for the first three days and then 0.1 mL of 5 mg/mL for the next 2–7 days), alternating between right and left flanks for a total of five or ten days. Animals were housed in mesh-sided cages and exposed to continuous fan-generated air drafts with low humidity (35–40%). Litter matched controls were housed in normal conditions. Each experimental group contained 5 to 6 mice. Aqueous tear production was assessed by placing a phenol red cotton thread (Zone-Quick; FCI Ophthalmics) in the lateral canthus for 1 min and reported as millimeters of wetted thread. Fluorescein staining was done as previously described [1]. Eyes were rinsed with PBS after induction of anesthesia. A drop (5 μL) of fluorescein solution (0.02%) was then added to the ocular surface, and corneal epithelial integrity examined using a slit lamp. Each experiment provided up to 12 eyes per group for analysis.

### Fluorescence *in situ* hybridization (FISH)

Enucleated mouse eyes were fixed in paraformaldehyde (2%) for 1 h with shaking at room temperature (RT). Bacterial hybridization was performed using a universal 16S rRNA gene probe [Alexa488]-GCTGCCTCCCGTAGGAGT-[Alexa488] (Eurofins Genomics) as previously described [1, 2]. Briefly, eyes were washed in 80% EtOH, 95% EtOH, and then PBS for 10 min each with shaking at RT. Eyes were then placed in a hybridization buffer solution [NaCl (0.9 M), Tris-HCl (20 mM, pH 7.2) and SDS (0.01%)] and incubated at 55°C for 30 min. The probe was added to final concentration of 100 nM and incubated at 55°C overnight. Eyes were then transferred to wash buffer solution [NaCl (0.9 M) and Tris-HCl (20 mM, pH 7.2)] and washed 3 times for 10 min each with shaking at RT.

### Alkyne functionalized D-alanine labeling

Labeling of live bacteria using an alkyne functionalized D-alanine (alkDala) biorthogonal probe [25] on the ocular surface was done as previously described [1]. Enucleated eyes were incubated in a solution of alkDala (10 mM) in Dulbecco’s Modified Eagle Medium (DMEM) at 37°C for 2 h. Eyes were then transferred to pre-chilled 70% EtOH and fixed for 20 min at -20°C. After rinsing, eyes were permeabilized in PBS containing Triton-X100 (0.5%) for 10 min with shaking at RT, then washed 3 times for 5 min each in PBS containing Triton-X100 (0.1%) and BSA (3%) with shaking at RT. Eyes were then transferred to the Click-labeling cocktail [in PBS, TBTA (100 μM), CuSO_4_ (1 mM), sodium ascorbate (2 mM), 488 nm azide fluorophore (10 μM), BSA (0.1 mg/mL)] for 1 h with shaking at RT.

### Confocal microscopy

Murine eyeballs were imaged *ex vivo* as previously described [1, 10, 11]. Briefly, eyes were fixed to a 12 mm glass coverslip with cyanoacrylate glue. The coverslip with eyeball was placed in a 47 mm Petri dish and filled with PBS to cover the eyeball completely. Confocal imaging was performed using an Olympus FV1000 confocal microscope. A 488 nm laser was used for detection of bacteria labeled with alkDala or FISH, and a 559 nm laser used for detection of red-fluorescent cellular membranes when appropriate, and a 635 nm laser used to obtain corneal reflectance (excitation and emission at the same wavelength) when mice without fluorescent membranes were used. At least four randomly-chosen fields were imaged for each eye (each field ~ 0.04 mm^2^) and fields examined from the corneal surface through the entire epithelium in 0.5 μm steps. The total number of bacteria detected was then expressed as bacteria per field of view. Three-dimensional images were reconstructed from z-stacks using IMARIS software (Bitplane) which was also used to quantify detected bacteria.

### Statistical analysis

Numerical data were expressed as a median with lower and upper quartiles for each experimental group. Statistical significance of differences between was determined using the Mann-Whitney *U* test (2 groups) or the Kruskal–Wallis test with Dunn’s multiple comparison test (3 groups). *P* values of < 0.05 were considered significant. *In vivo* EDE experiments were repeated at least once.
